# Supplementary material for: Opportunities for Improving Waterlogging Tolerance in Cereal Crops—Physiological Traits and Genetic Mechanisms
Source: Plants (Basel). 2021 Jul 29;10(8):1560. doi: 10.3390/plants10081560 (PMC8401455; doi:10.3390/plants10081560)
Supplement: Supplementary file 1 [file plants-10-01560-s001.zip › plants-1288767-supplementary.pdf]

Table S1. Summary of QTLs associated with waterlogging tolerance in maize, rice, wheat, and barley.

Species	Chr	QTL	Trait	LOD [a]	PVE (%) [b]	Population [c]	Population size	Population type	Reference
**Maize**	1	*R_GY*	Grain yield	6.4	5.0	**CAWL-46-3-1** ×CML311-2-1-3	211	RILs	[166]
**Maize**	1	*Qaer1.02-1.03*	Aerenchyma formation	4.1	10.8	B64 × ***Zea nicaraguensis***	186	F_2	[112]
**Maize**	1	*Qaer1.07*	Aerenchyma formation	5.3	11.7	B64 × ***Zea nicaraguensis***	186	F_2	[112]
**Maize**	1	*rl1*	Root length	2.74	5.1	HZ32 × **K12**	288	$F_{2:3}$	[43]
**Maize**	1	*rdw1*	Root dry weight	2.89	4.4	HZ32 × **K12**	288	$F_{2:3}$	[43]
**Maize**	2	*Qaer2.06*	Aerenchyma formation	4.1	9.1	B73 × ***Zea luxurians***	195	F_2	[167]
**Maize**	3	*R_GY*	Grain yield	6.1	4.2	**CAWL-46-3-1** ×CML311-2-1-3	211	RILs	[166]
**Maize**	3	*Qaer3.10*	Aerenchyma formation	1.8	6.3	**B73** × *Zea luxurians*	195	F_2	[167]
**Maize**	3	*Qarf3.07-3.08*	Adventitious root formation	3.2	10.0	B64 × **Na4**	110	F_2	[115]
**Maize**	3	*tdw3-3*	Total dry weight	2.64	12.2	HZ32 × **K12**	288	$F_{2:3}$	[43]
**Maize**	3	*tdw3-4*	Total dry weight	2.77	12.8	HZ32 × **K12**	288	$F_{2:3}$	[43]
**Maize**	3	*ph3*	Plant height	2.66	5.4	HZ32 × **K12**	288	$F_{2:3}$	[43]
**Maize**	3	*sdw3-2*	Shoot dry weight	2.68	5.6	HZ32 × **K12**	288	$F_{2:3}$	[43]
**Maize**	3	*rl3-1*	Root length	2.80	5.3	HZ32 × **K12**	288	$F_{2:3}$	[43]
**Maize**	3	*rl3-2*	Root length	3.49	6.7	HZ32 × **K12**	288	$F_{2:3}$	[43]
**Maize**	3	*rl3-3*	Root length	2.5	6.1	HZ32 × **K12**	288	$F_{2:3}$	[43]
**Maize**	3	*rdw3*	Root dry weight	2.67	5.2	HZ32 × **K12**	288	$F_{2:3}$	[43]
**Maize**	4	–	Leaf injury	11.9-25.5	29.0-49.0	***Zea.nicaraguensis*** × Mi29	652	BC_3F_4	[113]
**Maize**	4	*Qarf4.07*	Adventitious root formation	4.1	9.0	B64 × ***Zea mays ssp. Huehuetenangensis***	186	F_2	[56]
**Maize**	4	*sdw4-3*	Shoot dry weight	2.60	5.1	HZ32 × **K12**	288	$F_{2:3}$	[43]
**Maize**	5	*R_GY*	Grain yield	4.5	8.0	**CAWL-46-3-1** ×CML311-2-1-3	211	RILs	[166]
**Maize**	5	-	Root and shoot fresh and dry weight	3.02-6.10	6.32-12.01	**HZ32**×K12	180	BC_2F_2	[116]
**Maize**	5	*Qaer5.05-5.06*	Aerenchyma formation	2.6	4.2	B73 × ***Zea luxurians***	195	F_2	[167]
**Maize**	5	*Qaer5.09*	Aerenchyma formation	4.6	10.9	**B64** × *Zea nicaraguensis*	186	F_2	[112]
**Maize**	6	*sdw6-1*	Shoot dry weight	2.96	5.4	**HZ32** × K12	288	$F_{2:3}$	[43]
**Maize**	6	*Subtol6*	Mean leaf senescence score	-	22.0	**Mo18W**×B73	166	RILs	[114]
**Maize**	7	*rl7-3*	Root length	2.68	4.0	HZ32 × **K12**	288	$F_{2:3}$	[43]
**Maize**	7	*rl7-5*	Root length	2.79	5.5	**HZ32** × K12	288	$F_{2:3}$	[43]
**Maize**	7	*R_GY*	Grain yield	11.6	6.1	CAWL-46-3-1 **×CML311-2-1-3**	211	RILs	[166]
**Maize**	7	*Qarf7.04-7.05*	Adventitious root formation	5.1	21.0	B64 × **Na4**	110	F_2	[115]
**Maize**	8	*Qaer8.06-7*	Aerenchyma formation	4.5	11.6	**B64** × *Zea nicaraguensis*	186	F_2	[112]

**Maize**	8	*Qarf8.05*	Adventitious root formation	4.9	5.0	B64 × **Na4**	110	F_2	[115]
**Maize**	8	*Qarf8.03*	Adventitious root formation	4.0	10.0	B64 × ***Zea mays ssp. Huehuetenangensis***	186	F_2	[56]
**Maize**	8	*Qarf8.05*	Adventitious root formation	7.0	25.0	B64 × ***Zea mays ssp. Huehuetenangensis***	186	F_2	[56]
**Maize**	9	*Qaer9.07-9.08*	Aerenchyma formation	1.5	4.5	B73 × ***Zea luxurians***	195	F_2	[167]
**Maize**	9	*sdw9-4*	Shoot dry weight	7.03	20.8	HZ32 × **K12**	288	$F_{2:3}$	[43]
**Maize**	9	*tdw9-2*	Total dry weight	5.92	31.7	**HZ32** × K12	288	$F_{2:3}$	[43]
**Maize**	9	*tdw9-3*	Total dry weight	5.91	30.7	**HZ32** × K12	288	$F_{2:3}$	[43]
**Maize**	10	*R_GY*	Grain yield	5.4	3.6	CAWL-46-3-1 ×**CML311-2-1-3**	211	RILs	[166]
**Maize**	10	*Qaer10.04*	Aerenchyma formation	1.8	3.7	**B73** × *Zea luxurians*	195	F_2	[167]
**Maize**	10	*ph10-1*	Plant height	3.06	7.1	HZ32 × **K12**	288	$F_{2:3}$	[43]
**Maize**	10	*ph10-2*	Plant height	3.23	5.2	HZ32 × **K12**	288	$F_{2:3}$	[43]
**Maize**	10	*rl10*	Root length	2.77	5.6	**HZ32** × K12	288	$F_{2:3}$	[43]
**Rice**	1	$qTIL1^{C9285}$	Total internode elongation length	4.43	22.0	**C9285** × T65	94	F_2	[118]
**Rice**	1	$qTIL1^{T65}$	Total internode elongation length	3.86	20.0	W0120 × **T65**	94	F_2	[118]
**Rice**	3	$qLEI3^{C9285}$	Lowest elongated internode	3.14	14.0	**C9285** × T65	94	F_2	[118]
**Rice**	8	$qTIL8^{T65}$	Total internode elongation length	3.36	17.0	W0120 × **T65**	94	F_2	[118]
**Rice**	9	*Sub1*	Green leaf recovery	36.0	69.0	**IR40931-26** × PI543851	169	F_2	[120]
**Rice**	12	$qTIL12^{C9285}$	Total internode elongation length	6.19	27.0	**C9285** × T65	94	F_2	[118]
**Rice**	12	$qTIL12^{W0120}$	Total internode elongation length	5.89	36.0	**W0120** × T65	94	F_2	[118]
**Rice**	12	$qNEI12^{C9285}$	Number of elongated internodes	6.25	27.0	**C9285** × T65	94	F_2	[118]
**Rice**	12	$qNEI12^{W0120}$	Number of elongated internodes	4.47	27.0	**W0120** × T65	94	F_2	[118]
**Rice**	12	$qLEI12^{C9285}$	Lowest elongated internode	7.75	36.0	**C9285** × T65	94	F_2	[118]
**Rice**	12	$qLEI12^{W0120}$	Lowest elongated internode	5.63	26.9	**W0120** × T65	94	F_2	[118]
**Wheat**	1BL	*QRfbio.ua-1B-WGH*	Root fresh biomass	6.6	22.0	**USG3209** × Jaypee	130	RILs	[122]
**Wheat**	1BL	*QRdbio.ua-1B-WGH*	Root dry biomass	3.7	18.0	**USG3209** × Jaypee	130	RILs	[122]
**Wheat**	1BL	*QSfbio.ua-1B-WGH*	Shoot fresh biomass	6.7	27.0	**USG3209** × Jaypee	130	RILs	[122]
**Wheat**	1BL	*QSpadpost.ua-1B-WF*	Chlorophyll content	4.8	32.0	**USG3209** × Jaypee	130	RILs	[122]
**Wheat**	2B	–	Root/shoot dry weight	3.00-8.29	9.5-23.3	**W7984** × Opata 85	112	RILs	[124]
**Wheat**	6B	–	Survival rate	3.45	10.3	**W7984** × Opata 85	112	RILs	[124]

**Wheat**	6D	*QRlength.ua-6D-WGH*	Root length	3.8	8.0	USG3209 × **Jaypee**	130	RILs	[122]
**Wheat**	6D	*QSdbio.us-6D-WGH*	Shoot dry biomass	3.6	6.0	**USG3209** × Jaypee	130	RILs	[122]
**Wheat**	6D	*QSpadpost.ua-6D-WF*	Chlorophyll content	3.2	6.0	**USG3209** × Jaypee	130	RILs	[122]
**Wheat**	7A	*GRI-7A*	Germination rate index	2.93-7.64	11.4-23.9	W7984 × **Opata 85**	112	RILs	[124]
**Wheat**	7B	–	Plant height index<br>Survival rate	2.97-4.27<br>4.66	8.7-12.1<br>13.6	**W7984** × Opata 85	112	RILs	[124]
**Barley**	1H	*yfy2.2-2*	Leaf yellowing	2.77	5.0	**Yerong** × Franklin	177	DH lines	[126]
**Barley**	1H	*tfy1.1-3*	Leaf yellowing	2.75	7.1	TX9425 × **Franklin**	177	DH lines	[126]
**Barley**	2H	*KWw1.1*<br>*KWw1.2*	Kernel weight	4.48<br>7.34	6.82<br>16.59	Yerong × **Franklin**	156	DH lines	[125]
**Barley**	2H	*KWw2.1*<br>*KWw2.2*	Kernel weight	9.10<br>3.94	27.35<br>6.78	Yerong × **Franklin**	156	DH lines	[125]
**Barley**	2H	*SLw2.1*<br>*SLw2.2*	Spike length	11.16<br>8.74	17.44<br>13.05	Yerong × **Franklin**	156	DH lines	[125]
**Barley**	2H	*GSw1.1*<br>*GSw2.1*	Grains per spike	11.53<br>5.63	35.35<br>55.34	**Yerong** × Franklin	156	DH lines	[125]
**Barley**	2H	*GSw1.2*<br>*GSw2.2*	Grains per spike	6.29<br>4.76	12.18<br>8.22	**Yerong** × Franklin	156	DH lines	[125]
**Barley**	2H	*GYw1.1*	Grain yield	2.85	4.74	**Yerong** × Franklin	156	DH lines	[125]
**Barley**	2H	*tfy1.1-1*	Leaf chlorosis	9.21	23.3	TX9425 × **Franklin**	92	DH lines	[126]
**Barley**	2H	*tfsur-1*	Survival rate	3.29	19.0	TX9425 × **Franklin**	92	DH lines	[126]
**Barley**	2H	*tfsur-2*	Survival rate	2.75	13.2	**TX9425** × Franklin	92	DH lines	[126]
**Barley**	2H	*yfsur-1*	Plant survival	3.15	7.1	**Yerong** × Franklin	177	DH lines	[126]
**Barley**	2H	*QWL.YeFr.2H.1*<br>*QWL.YeFr.2H.2*	Survival rate	4.62<br>10.21	8.4<br>17.2	Yerong ×**Franklin**	177	DH lines	[104]
**Barley**	2H	-	Yellow leaf percentage	2.95-10.21	4.0-17.2	Yerong × **Franklin**	177	DH lines	[104]
**Barley**	2H	*QWI.YyFr.2H*	Plant healthiness	18.68	30.1	**YYXT** × Franklin	172	DH lines	[127]
**Barley**	3H	SLw2.3	Spike length	7.74	14.37	Yerong × **Franklin**	156	DH lines	[125]
**Barley**	3H	*tfy1.2-1*<br>*tfy2.1-1*	Leaf chlorosis	7.31<br>9.28	36.0<br>34.1	TX9425 × **Franklin**	92	DH lines	[126]
**Barley**	3H	*tfy1.1-2*	Leaf chlorosis	7.31	36.0	TX9425 × **Franklin**	92	DH lines	[126]
**Barley**	3H	*yfy2.1-2*<br>*yfy2.2-1*	Leaf yellowing	6.41<br>4.5	11.9<br>9.5	Yerong × **Franklin**	177	DH lines	[126]
**Barley**	3H	*QWI.YyFr.3H*	Plant healthiness	10.94	15.7	YYXT × **Franklin**	172	DH lines	[127]
**Barley**	3H	*QWL.YeFr.3H*	Survival rate	4.83	6.8	Yerong ×**Franklin**	177	DH lines	[104]

**Barley**	4H	-	Yellow leaf percentage	3.78-16.51	6.7-26.7	Yerong × **Franklin**	177	DH lines	[104]
**Barley**	4H	*QWL.YeFr.4H*	Survival rate	14.48	23.9	Yerong × **Franklin**	177	DH lines	[104]
**Barley**	4H	QTL-AER	Aerenchyma formation	51.4	76.8	**TAM407227** × Franklin	163	DH lines	[108]
**Barley**	4H	*QTL-WL-4H*	Waterlogging tolerance	19.2	34.6	**TAM407227** × Franklin	163	DH lines	[108]
**Barley**	4H	–	Root porosity<br>Aerenchyma formation	6.4<br>9.4	25.6<br>44	**Yerong** × Franklin	177	DH lines	[111]
**Barley**	4H	*yfy2.1-3*<br>*yfy2.2-3*	Leaf yellowing	9.25<br>10.37	18.5<br>22.4	Yerong × **Franklin**	177	DH lines	[126]
**Barley**	4H	–	Root porosity	12.1-13.5	35.7-39.0	**YYXT** × Franklin	126	DH lines	[110]
**Barley**	4H	*tfmas*	Biomass reduction	2.75	16.3	TX9425 × **Franklin**	92	DH lines	[126]
**Barley**	4H	*QWI.YyFr.4H*	Plant healthiness	5.25	7.0	YYXT × **Franklin**	172	DH lines	[127]
**Barley**	5H	*yfy1.1-2*	Leaf yellowing	3.94	7.6	**Yerong** × Franklin	177	DH lines	[126]
**Barley**	5H	*yfsur-2*	Leaf yellowing	5.05	13.1	Yerong × **Franklin**	177	DH lines	[126]
**Barley**	6H	*PHw2.1*	Plant height	5.52	9.74	**Yerong** × Franklin	156	DH lines	[125]
**Barley**	6H	-	Root porosity	3.7	10.2	**Yerong** × Franklin	177	DH lines	[111]
**Barley**	6H	*QWI.YyFr.6H*	Plant healthiness	4.72	6.2	YYXT × **Franklin**	172	DH lines	[127]
**Barley**	6H	-	Yellow leaf percentage	3.72-7.56	6.7-11.4	Yerong × **Franklin**	177	DH lines	[104]
**Barley**	6H	QTL-WL-6H	Waterlogging tolerance	4.4	6.3	**TAM407227** × Franklin	163	DH lines	[108]
**Barley**	7H	QTL-WL-7H	Waterlogging tolerance	3.7	5.3	**TAM407227** × Franklin	163	DH lines	[108]
**Barley**	7H	*yfy2.1-1*	Leaf yellowing	3.72	6.7	Yerong × **Franklin**	177	DH lines	[126]
**Barley**	7H	GYw1.2	Grain yield	7.45	30.43	**Yerong** × Franklin	156	DH lines	[125]
**Barley**	7H	*SPw1.1*	Spike per plant	3.31	8.13	Yerong × **Franklin**	156	DH lines	[125]
**Barley**	7H	*GSw1.3*	Grains per spike	7.35	9.06	**Yerong** × Franklin	156	DH lines	[125]

Table S2. The detailed information of syntenic candidate genes in barley, wheat, rice, and maize.

Gene	Annotation	Barley	Wheat A genome	Wheat B genome	Wheat D genome	Rice	Maize
HRE2(At2g47520)	Ethylene-responsive transcription	ERF1 (HORVU.MOREX.r3.1HG0060770.1)	ERF1 (TraesCS1A02G231200.1)	ERF1 (TraesCS1B02G243200.1)	ERF1 (TraesCS1D02G230900.1)	ERF1 (Os05t0361700-01)	ERF1 (Zm00001d037941_P001)
ZmEREB180	Ethylene-responsive transcription	ERF2 (HORVU.MOREX.r3.4HG0405650)	ERF2 (TraesCS4A02G005600.1)	ERF2 (TraesCS4B02G299400.1)	ERF2 (TraesCS4D02G298400.1)	ERF2 (Os03t0183000)	ERF2 (Zm00001d027925_P001)
SNORKEL1	Ethylene-responsive transcription	ERF3 (HORVU.MOREX.r3.5HG0481240)	ERF3 (TraesCS5A02G215900.1)	ERF3 (TraesCS5B02G214400.1)	ERF3 (TraesCS5D02G223700.1)	ERF3 (Os09t0434500-01)	ERF3 (Zm00001d020595_P001)
GRMZM2G053503	Ethylene-responsive transcription	ERF4 (HORVU.MOREX.r3.5HG0497970.1)	ERF4 (TraesCS5A02G314600.1)	ERF4 (TraesCS5B02G315500.1)	ERF4 (TraesCS5D02G320800.1)	ERF4 (Os01t0313300-01)	ERF4 (Zm00001d009103_P001)
Sub1A	Ethylene-responsive transcription	N	ERF5 (TraesCS5A02G174700.1)	N	N	ERF5.1 (Os09t0287000)	N
Sub1B	Ethylene-responsive transcription	N	ERF5 (TraesCS5A02G174700.1)	N	N	ERF5.1 (Os09t0287000-01)	ERF5.1 (Zm00001d052087_P001)
Sub1C	Ethylene-responsive transcription	N	ERF5 (TraesCS5A02G174700.1)	N	N	ERF5.2 (Os09t0286600)	ERF5.2 (Zm00001d032095_P001)
SNORKEL2	Ethylene-responsive transcription	N	ERF6 (TraesCS7A02G120100.1)	ERF6 (TraesCS7B02G018400.1)	ERF6 (TraesCS7D02G118000.1)	ERF6 (Os12t0603300-01)	ERF6.1 (Zm00001d036251_P001), ERF6.2 (Zm00001d045262_P001)
HRE1(At1g72360)	Ethylene-responsive transcription	ERF7 (HORVU.MOREX.r3.7HG0661060.1)	ERF7 (TraesCS7A02G158000.1)	ERF7 (TraesCS7B02G062200.2)	ERF7 (TraesCS7D02G158500.2)	ERF7 (Os06t0194000-02)	ERF7 (Zm00001d045044_P001)
RAP2.2(At3g14230)	Ethylene-responsive transcription	ERF7 (HORVU.MOREX.r3.7HG0661060.1)	ERF7 (TraesCS7A02G158000.1)	ERF7 (TraesCS7B02G062200.1)	ERF7 (TraesCS7D02G158500.1)	ERF7 (Os06t0194000-01)	ERF7 (Zm00001d045044_P001)
RAP2.12(At1g53910)	Ethylene-responsive transcription	ERF7 (HORVU.MOREX.r3.7HG0661060.1)	ERF7 (TraesCS7A02G158000.1)	ERF7 (TraesCS7B02G062200.1)	ERF7 (TraesCS7D02G158500.1)	ERF7 (Os06t0194000-01)	ERF7 (Zm00001d045044_P001)
WRK45	WRKY transcription	WRKY1 (HORVU.MOREX.r3.2HG0096750.1)	WRKY1 (TraesCS2A02G011000.1)	WRKY1 (TraesCS2B02G010500.1)	WRKY1 (TraesCS2D02G011700.1)	WRKY2 (Os11t0490900-01)	WRKY1 (Zm00001d007329_P001)
WRK40	WRKY transcription	WRKY2 (HORVU.MOREX.r3.6HG0568570.1)	WRKY2 (TraesCS6A02G146900.1)	WRKY2 (TraesCS6B02G175100.2)	WRKY2 (TraesCS6D02G136200.1)	WRKY1 (Os02t0181300-01)	WRKY2 (Zm00001d015515_P001)
GRMZM2G416632	Glutathione S-transferase	GST1 (HORVU.MOREX.r3.5HG0487860.1)	GST1 (TraesCS5A02G247500.2)	GST1 (TraesCS5B02G244700.1)	GST1 (TraesCS5D02G254000.1)	GST1 (Os09t0467200-01)	GST1 (Zm00001d020780_P001; GST23)
GRMZM2G300965	Respiratory burst oxidase, putative	RBOH1 (HORVU.MOREX.r3.5HG0444960.1)	RBOH1 (TraesCS5A02G093600.1)	RBOH1 (TraesCS5B02G099700.1)	RBOH1 (TraesCS5D02G105900.1)	RBOH1 (Os12t0541300-01)	RBOH1 (Zm00001d023859_P001)
GRMZM2G055704	Heavy metal transport/detoxification superfamily protein	HMS1 (HORVU.MOREX.r3.4HG0410950.1)	HMS1 (TraesCS5A02G492100.1)	HMS1 (TraesCS4B02G319800.1)	N	HMS1 (Os03t0126700-01)	HMS1 (Zm00001d027454_P001)
